# Suicidal Behavior: Links Between Alcohol Use Disorder and Acute Use of Alcohol

**DOI:** 10.35946/arcr.v40.1.02

**Published:** 2019-09-13

**Authors:** Kenneth R. Conner, Courtney L. Bagge

**Affiliations:** Kenneth R. Conner, Psy.D., M.P.H., is a professor in the Departments of Emergency Medicine and Psychiatry, University of Rochester Medical Center, Rochester, New York. Courtney L. Bagge, Ph.D., is an associate professor in the Department of Psychiatry and Human Behavior, University of Mississippi Medical Center, Jackson, Mississippi

**Keywords:** alcohol consumption, alcohol use disorder, intoxication, suicide, suicide attempt

## Abstract

Research on associations of suicidal behavior, including suicide and suicide attempt, with alcohol use disorder (AUD) and acute use of alcohol (AUA) are discussed, with an emphasis on data from meta-analyses. Based on psychological autopsy investigations, results indicate that AUD is prevalent among individuals who die by suicide. Results also indicate that AUD is a potent risk factor for suicidal behavior. Risk estimates are higher for individuals with AUD in treatment settings, when compared to individuals in the community who have AUD. Also, although rates of suicide and prevalence of AUD remain higher in men, they have increased more among women in recent decades. Based on postmortem blood alcohol concentrations, AUA was commonly present among those who died by suicide. AUA is a potent proximal risk factor for suicidal behavior, and the risk increases with the amount of alcohol consumed, consistent with a dose-response relationship. Research indicates that AUA increases risk for suicidal behavior by lowering inhibition and promoting suicidal thoughts. There is support for policies that serve to reduce alcohol availability in populations with high rates of AUD and suicide, that promote AUD treatment, and that defer suicide risk assessments in intoxicated patients to allow the blood alcohol concentration to decrease.

## Introduction

Suicide claims more than 800,000 lives each year worldwide and is the second-leading cause of death among people ages 15 to 29.^1^ For every suicide, at least 20 nonlethal suicide attempts have occurred, primarily by attempted overdose. These attempts are a leading cause of hospitalizations from injury and a potent risk factor for eventual suicide. Therefore, examination of suicide and suicide attempt is a critical focus for injury research and prevention efforts. Alcohol use may confer risk for these outcomes proximally through the acute use of alcohol (AUA), which has been defined as the use of alcohol within 3 hours or within 6 hours of suicidal behavior, or as any blood alcohol concentration (BAC) in an individual who attempted suicide or died by suicide.^2^ Alcohol use may also confer risk for suicidal behavior more distally through chronic effects, including those manifested in alcohol use disorder (AUD).^3^ Accordingly, the role of AUA and AUD in suicidal behavior, including suicide and suicide attempt, is discussed.

## AUD and Suicidal Behavior

Estimates of risk for suicide associated with the presence of AUD were provided by meta-analyses of postmortem case-control studies (*N* = 35, *OR* = 3.68, 95% confidence interval [CI: 1.99, 6.82]),^4^ and studies using mixed designs, including case-control and cohort studies (*N* = 31, *OR* = 2.59, 95% CI [1.95, 3.23]).^5^ The latter study also produced an estimate of risk for suicide attempt associated with AUD (*OR* = 3.13, 95% CI [2.45, 3.81]). These meta-analyses suggest that the odds of suicidal behavior are about three times higher among individuals with AUD compared to those without AUD. Higher risk estimates were produced in a meta-analysis of suicide based on cohort studies of treated patients with AUD (*N* = 17),^6^ which is attributable to its examination of clinical populations with more severe symptoms.

AUD is the second-most commonly identified mental disorder among suicide decedents worldwide (the most common is mood disorder),^7^ suggesting that AUD is a major contributor to population-level rates of suicide.^8^ However, the percentage of suicide decedents who had AUD, as identified in psychological autopsy studies, has ranged widely, from a low of 7% in the National Psychological Autopsy Study in China^9^ to a high of 61% in a report from Estonia.^10^ Key reasons that AUD is a major risk factor for suicide include its role in contributing to substance-induced depressive episodes, disruptions in interpersonal relationships (e.g., breakups), and repeated exposure to alcohol intoxication.^11^

Although two of the aforementioned meta-analyses did not identify gender differences in the risk for suicide associated with AUD,^4^,^5^ the meta-analysis of cohorts of patients with AUD produced a higher risk estimate for suicide among women with AUD (standardized mortality risk [SMR] = 16.39, 95% CI [10.66, 25.19]) than among men with AUD (SMR = 8.75, 95% CI [6.35, 12.06]).^6^ This result suggests that women who receive AUD treatment have about a 16-fold risk for suicide compared with women in the general population, whereas men who have received AUD treatment have approximately a 9-fold risk for suicide compared with men in the general population. The need to examine gender differences in AUD-related risk is underscored by trends in recent decades in the United States, which show substantially greater increases among women than men in rates of suicide^12^ and prevalence of AUD.^3^

## AUA and Suicidal Behavior

In the United States, approximately 36% of male and 29% of female suicide decedents ages 18 and older have a postmortem BAC of 0.001 g/dL or more, and 24% of males and 17% of females have BAC levels that exceed 0.08 g/dL, the U.S. national legal limit for drinking and driving.^13^ To estimate risk for suicidal behavior associated with AUA, controlled studies have compared AUA that occurred before suicidal behavior to AUA that occurred within the same subjects during a lower-risk control period (case-crossover design) and to AUA that occurred during a comparable period of time in a lower-risk control group (case-control design).^2^ A meta-analysis of such reports showed that although risk for suicide attempt increases at low levels of AUA (*OR* = 2.71, 95% CI [1.56, 4.71]), risk increases markedly at high levels of AUA (*OR* = 37.18, 95% CI [17.38, 79.53]), as defined by a BAC of more than 0.10 g/dL, which is consistent with a dose-response relationship. A rigorous, controlled study of AUA and suicide by firearm also demonstrated a dose-response relationship between the amounts of alcohol consumed and risk.^14^ Such data provide a strong empirical rationale for the common clinical practice of holding intoxicated, suicidal patients in emergency settings to allow for a drop in BAC before assessing suicidal risk and considering discharge.

A seminal review posited several mechanisms by which AUA may increase risk for suicidal behavior, including alcohol-related increases in psychological distress, depressed mood, aggressiveness, and impulsivity.^15^ The role of alcohol in cognitive constriction, a narrowing of attention to one’s present emotional state and circumstances, is another likely mechanism.^16^ Recent research has shown that during the 24-hour period preceding a suicide attempt, AUA in a given hour is associated with increased intensity of suicidal ideation in the next hour.^17^ Research has also shown that AUA is associated with a rapid transition from acute suicidal impulse to action,^18^ suggesting that the role of AUA in promoting suicidal ideation and disinhibition is a mechanism of risk for suicidal behavior. A link between AUA and the use of firearms, the most lethal form of self-injury and the most common method of suicide in the United States, is also critical to consider.^19^ Data show that alcohol intoxication is most commonly present in suicide by firearm among young adult and middle-aged men.^20^ This research indicates the importance of focusing suicide by firearm prevention efforts on this segment of the U.S. population.

There is heterogeneity in the motives for AUA preceding suicidal behavior, with approximately a quarter to a third of individuals who drank acutely before a suicide attempt reporting they did so in an effort to facilitate the act of suicide by seeking to build courage, numb fears, or anesthetize the pain of dying.^21^,^22^ Also, for suicide risk, AUA may act synergistically with other substances, as demonstrated by a report showing that co-ingestion of AUA and acute use of other central nervous system depressants (sedatives, anxiolytics, or opioids) increased risk for a suicide attempt, with an *OR* of 8.76 (95% CI [1.02, 75.44]), when compared with AUA only (*OR* = 4.07, 95% CI [2.06, 8.02]) or when compared with acute use of other central nervous system depressants only (*OR* = 3.01, 95% CI [1.09, 8.31]).^23^

## Implications and Future Directions

Interventions that serve to decrease alcohol use and AUD in general populations through policies such as alcohol taxes or restrictions on alcohol availability^24^ may be expected to have the greatest effect on suicide rates in countries with high rates of AUD and alcohol consumption per capita. Illustrating this idea, a national campaign in Russia to reduce alcohol availability led to reduced rates of suicide, which increased to preintervention levels following cessation of the campaign.^25^ Moreover, the temporary decline in suicide rates appeared to be attributed to the decrease in suicides associated with BAC levels of more than 0.15 g/dL.^25^ See the report by Xuan and colleagues for a comprehensive review of the literature on alcohol policies and suicide.^24^

Clinical policy interventions targeting AUD also have the potential to affect suicide rates in health systems that have high rates of AUD and suicide. AUD is a potent risk factor for suicide and the second-most common mental disorder (the most common is depression) among U.S. veterans receiving treatment in the Veterans Health Administration (VHA) who eventually die by suicide.^26^ Moreover, initiation of AUD treatment has been shown to lower prospective risk for suicide attempt among veterans in VHA treatment,^27^ suggesting the importance of AUD screening and suicide prevention efforts during treatment for AUD.

Assessments of the role of AUA in suicide attempts should begin with establishing if AUA occurred and estimating the amount of alcohol consumed. Assessments may include determining a patient’s motivation for drinking before the attempt and a collaborative chain analysis with the patient.^28^ Chain analysis is a retrospective method for determining the sequence of events, thoughts (e.g., suicide premeditation and drinking motivations), and behaviors (e.g., drinking) that led up to a suicidal act. The information learned from a chain analysis can be used to develop a personalized distress safety plan that highlights high-risk periods and warning signs, and to devise strategies for avoiding alcohol.^17^ Overall, the goal of the plan is to prevent escalation of suicidal risk in the context of AUA.

Future research directions include the study of real-time interventions via mobile applications, which could potentially coach individuals on adaptive strategies for suicidal thoughts, urges to drink, or distressing experiences. Another future direction is to accelerate research on pharmacological interventions that target individuals at risk for alcohol-related suicidal behavior.
